# Outcomes of Primary Care for People With Dementia: What’s Important?

**DOI:** 10.1093/geroni/igaa057.533

**Published:** 2020-12-16

**Authors:** Laura Wray, Bonnie Vest, Laura Brady, Christina Vair, Gregory Beehler, John McCarten

**Affiliations:** 1 Veterans Health Administration, Buffalo, New York, United States; 2 University at Buffalo, Buffalo, New York, United States; 3 SUNY University at Buffalo, Buffalo, New York, United States; 4 Department of Veterans Affairs - Salisbury VA Health Care System, Salisbury, North Carolina, United States; 5 Buffalo VAMC, Buffalo, New York, United States; 6 Minneapolis VA Medical Center, Minneapolis, Minnesota, United States

## Abstract

People with dementia (PWD) typically receive most of their healthcare in primary care (PC), but neurocognitive disorders can be challenging to recognize, assess, and manage in that setting. As a result, cognitive impairment in older adults is often missed or not addressed until later stages. The result is poor management of comorbid health conditions, increased healthcare utilization, and negative outcomes for the patient and family. Further, strategies for improvement and barriers to high quality PC for PWD have received limited attention. To improve PC for PWD, it is essential to understand what care outcomes should be targeted. To address this gap, we used a qualitative approach to examine potential outcomes of PC from the perspectives of older adults, family caregivers, primary care teams, and geriatrics specialists (n=79) from two Veterans Health Administration healthcare systems. Participants were interviewed individually or in focus groups. A directed content analysis based on the adapted Donabedian model was employed and expanded to fully capture transcript content. Three main categories of outcomes were identified: Personhood (i.e., independence), Physical Health and Safety, and Quality of Life. Regardless of participant type, respondents focused on similar desired outcomes and, notably, identified outcomes as important for both patients and their broader social context (i.e., caregivers, family). Discussion will: show how findings align with work conducted in specialty and residential care; describe how challenges to attaining these outcomes in PC can be overcome; and, challenge cognitive screening recommendations for PC that are based primarily on risk/benefit analysis of medication-focused outcomes.

